# Synthesis of Au, Ag, and Au–Ag Bimetallic Nanoparticles Using *Pulicaria undulata* Extract and Their Catalytic Activity for the Reduction of 4-Nitrophenol

**DOI:** 10.3390/nano10091885

**Published:** 2020-09-20

**Authors:** Merajuddin Khan, Khaleel Al-hamoud, Zainab Liaqat, Mohammed Rafi Shaik, Syed Farooq Adil, Mufsir Kuniyil, Hamad Z. Alkhathlan, Abdulrahman Al-Warthan, Mohammed Rafiq H. Siddiqui, Mihail Mondeshki, Wolfgang Tremel, Mujeeb Khan, Muhammad Nawaz Tahir

**Affiliations:** 1 Department of Chemistry, College of Science, King Saud University, P.O. Box 2455, Riyadh 11451, Saudi Arabia; mkhan3@ksu.edu.sa (M.K.); khaleel727244@gmail.com (K.A.-h.); mrshaik@ksu.edu.sa (M.R.S.); sfadil@ksu.edu.sa (S.F.A.); mkuniyil@ksu.edu.sa (M.K.); khathlan@ksu.edu.sa (H.Z.A.); awarthan@ksu.edu.sa (A.A.-W.); rafiqs@ksu.edu.sa (M.R.H.S.); 2 Department Chemie, Johannes Gutenberg Universität of Mainz, Duesbergweg 10-14, D-55128 Mainz, Germany; lizainab@uni-mainz.de (Z.L.); mondeshk@uni-mainz.de (M.M.); 3 Department of Chemistry, King Fahd University of Petroleum & Minerals, P.O. Box 5048, Dhahran 31261, Saudi Arabia

**Keywords:** gold, silver, bimetallic, nanoparticles, plant extract, *Pulicaria undulata*, catalytic activity

## Abstract

Plant extract of *Pulicaria undulata* (L.) was used as both reducing agent and stabilizing ligand for the rapid and green synthesis of gold (Au), silver (Ag), and gold–silver (Au–Ag) bimetallic (phase segregated/alloy) nanoparticles (NPs). These nanoparticles with different morphologies were prepared in two hours by stirring corresponding metal precursors in the aqueous solution of the plant extracts at ambient temperature. To infer the role of concentration of plant extract on the composition and morphology of NPs, we designed two different sets of experiments, namely (i) low concentration (LC) and (ii) high concentration (HC) of plant extract. In the case of using low concentration of the plant extract, irregular shaped Au, Ag, or phase segregated Au–Ag bimetallic NPs were obtained, whereas the use of higher concentrations of the plant extract resulted in the formation of spherical Au, Ag, and Au–Ag alloy NPs. The as-prepared Au, Ag, and Au–Ag bimetallic NPs showed morphology and composition dependent catalytic activity for the reduction of 4-nitrophenol (4-NPh) to 4-aminophenol (4-APh) in the presence of NaBH_4_. The bimetallic Au–Ag alloy NPs showed the highest catalytic activity compared to all other NPs.

## 1. Introduction

Silver and gold nanoparticles (NPs) have been the focus of research because of their optical and plasmonic properties as well as their unique surface enhanced Raman spectroscopy (SERS) behavior [1,2]. These properties depend on particle composition, size, and morphology, which can be used for applications in diagnostics, as biosensors, and in catalysis [3,4]. The optical properties of the Au and Ag particles can be tuned efficiently by the size (isotropic) and aspect ratio (anisotropic) over a wide spectral range or composition (bimetallic), which leads to strongly enhanced catalytic and SERS behavior [5]. Much effort has been devoted to the synthesis of plasmonic metal NPs with different morphologies, and the demand for new synthetic methods is still increasing. Synthetic methods involve both physical techniques (spray pyrolysis, ball milling, sputtering, etc.) and chemical processes. The solution-based chemical methods are best suited for controlling the particle size and morphology because nucleation and growth steps can be easily monitored [6,7,8,9,10]. This requires different hazardous chemicals such as chemical reductants and capping agents to compensate the surface energy to stabilize these NPs in the nano-size regime. However, aqueous synthesis using different natural products such as sugars, ascorbic acid, and sodium citrate has been much explored [11,12,13], especially for the synthesis of metallicNPs. Although these NPs are biocompatible and widely used in different biological applications, there is still demand for the development of other methods that are low cost and easily scalable. In this contribution, the use of plant extracts, being renewable, easy to grow on a mass scale, and environmentally benign, have caught the attention of the researchers [14,15,16]. These renewable bio-resources have some advantages, as they minimize the use of surfactants and allow the replacement of organic solvents by water. Plant extracts have the potential for large scale production as they are easy to handle, abundant, and inexpensive. However, less control, longer reaction times, and higher temperatures have often hindered the growth of plant extract-based biosynthetic procedures when compared to the chemical synthesis of NPs [17].

A number of studies has appeared on the green synthesis of Ag and Au NPs using plant extracts as reducing agents [18,19]. Ganesan et al. reported the preparation of spherical shaped, ~10 nm sized Au NPs using an aqueous extract of *Acorus calamus* rhizome as the reducing agent [20]. Ahmad et al. demonstrated the active role of polyphenolic compounds present in aqueous extracts of *Elaeis guineensis* leaves in the synthesis of Au NPs [21]. Similarly, polyphenolic compounds of curcumin have also been exploited for the synthesis of high-quality silver NPs in aqueous medium. The hydrophobic curcumin was encapsulated in cyclodextrin to enhance its aqueous solubility [22]. Similarly, leaf extracts of three different plants including *Phyllanthus urinaria*, *Pouzolzia zeylanica*, and *Scoparia dulcis* have also been recently utilized to obtain Ag NPs [23]. There are, however, only a few studies and preparations of bimetallic particles with plant extracts [24]. Bimetallic Au–Ag NPs have been prepared with *Azadirachta indica* leaf broth [25], and Gopinath et al. prepared spherical Au–Ag NPs (average size 20 nm) using *Gloriosa superba* leaf extracts [26]. Recently, three types of Au–Ag bimetallic NPs were prepared by using leaf extracts of fenugreek, coriander, and soybean as reducing agents in a single-pot reduction process [27]. Similarly, Sharma et al. used an aqueous extract of clove buds to prepare Au–Ag bimetallic NPs [28]. Apart from this, other types of plant materials have also been used for similar purpose [29]. Here, we demonstrate the use of the water soluble portion of extract of *Pulicaria undulata* (L.) for the synthesis of Au, Ag, and Au–Ag bimetallic NPs. The morphology of the synthesized mono/bimetallic NPs is very much dependent on the concentration of plant extract. 

*P. undulata* is one of the most important plant species of the genus *Pulicaria* belonging to the family Asteraceae. *P. undulata* has been used in the treatment of several diseases by traditional and modern medicine [30,31]. *P. undulata* has been reported to contain several classes of phytomolecules and is rich in certain polyphenols that are known to play an important role in the biosynthesis of nanomaterials [32].

**Scheme 1:**
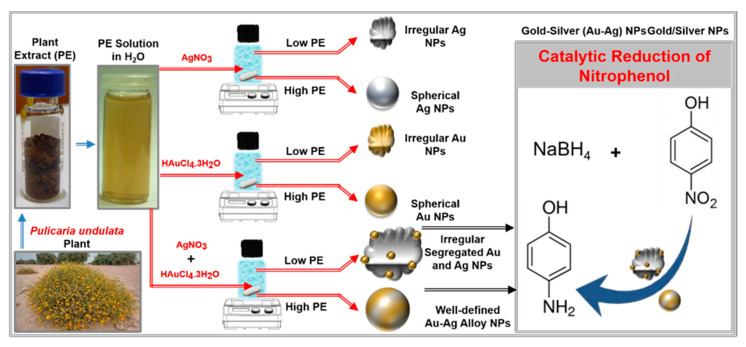
Schematic presentation of synthesis of Ag, Au, and Au–Ag bimetallic NPs using *P. undulata* plant extract and their catalytic activities.

Ag and Au NPs are used in organic synthesis as catalysts for oxidation, reduction, and cycloaddition reactions and as redox catalysts for the degradation of organic pollutants through electron shuttle effects between donor and acceptor molecules [33]. Nitrophenols are common organic pollutants in industrial and agricultural wastewaters. They are water soluble, stable, and therefore difficult to degrade. In addition, reduction of the nitro (–NO_2_) group to amine (–NH_2_) is a very important organic transformation used in many industrially important compounds. Therefore, the as-prepared Au, Ag, and Au–Ag bimetallic NPs in water were used as active catalysts for the model reduction of 4-nitrophenol (4-NPh) to 4-aminophenol (4-APh) (Scheme 1).

## 2. Materials and Methods

### 2.1. Materials

Hydrogen tetrachloroaurate (III) trihydrate (HAuCl_4_ × 3H_2_O, 99.9%, Aldrich, St. Louis, MO, USA), silver nitrate (AgNO_3_, 99%, Aldrich), and 4-nitrophenol (4-NPh, Aldrich, >99.5%) were used. Reagent grade solvents were used. Milli-Q water was obtained from a Milli-Q SP ultrapure water purification system from Nihon Millipore Ltd., Tokyo, Japan.

### 2.2. Preparation of Plant Extract (PE)

Extract from the aerial parts of *P. undulata* were prepared as described before [34]. Freshly collected plant of *P. undulata* were identified by a plant taxonomist from the herbarium division, College of Science, King Saud University. The aerial parts of fresh *P. undulata* were cut into small pieces and dried at room temperature for 15 days in the shade. The dried and powdered aerial parts (343 g) of *P. undulata* were extracted for 3 h with boiling water. The aqueous extract was cooled and filtered through a Whatman filter paper. After drying at 50 °C in vacuo in a rotary evaporator, 24.0 g of dark brown powdered water extract was obtained and used for the subsequent synthesis of metal particles. 

### 2.3. Preparation of Au and Ag Nanoparticles

Stock solutions of the metal precursors and PEs were prepared in Milli-Q water. For Au and Ag solutions, the stock solutions were prepared by taking 10 mmol of the respective metal precursors (HAuCl_4_ × 3H_2_O and AgNO_3_) in 10 mL of water. The PE stock solution was prepared by dissolving 10 mg of PE in 1 mL of Milli-Q water. Au, Ag, and Au–Ag NPs were prepared by taking 1 mL of the respective stock solutions and was mixed with either 0.2 mL of the PE solution (termed low concentration (LC) of PE) or 0.4 mL of PE stock (termed higher concentration (HC)) followed by addition of 10 mL of Milli-Q water in a small glass bottle. The solutions were stirred slowly with a magnetic stirrer at room temperature until no further color change occurred. 

For Au NPs, the color of the solution gradually changed from light yellow to purple within 2 h, which indicated the reduction of Au^3+^ to Au^0^. For Ag NPs the color of the solution changed from colorless to dark yellow within 2 h. The progress of the reaction was monitored in regular intervals by UV spectroscopy. 

### 2.4. Preparation of Au–Ag Alloy Nanoparticles

The Au–Ag bimetallic NPs were also prepared in the analogous fashion using 0.9 mL of the Au and 0.1 mL of the Ag stock solutions, keeping the concentration of PE constant for both sets of experiments as in the case of the monometallic counterparts. The color of the reaction mixture changed from light yellow to purple within ~2 h. The experiments for the preparation of Au, Ag, and Au–Ag alloy NPs were repeated to confirm the reproducibility of the reaction. All the reactions were performed by using a high concentration of plant extract using the same aforementioned procedure. The samples were characterized by UV and TEM analyses, and the results are provided in the Supplementary Information Figure S1. 

### 2.5. Characterization

UV spectroscopy was performed on a Lambda 35 UV–Vis spectrophotometer (PerkinElmer, Waltham, MA, USA) in quartz cuvettes using distilled water as the reference solvent. The samples were prepared by diluting 1 mL of the NP reaction solution (collected during and at the end of the reaction) in 9 mL of water. TEM and EDX analysis was carried out on a JEM 1101 transmission electron microscope (JEOL, Tokyo, Japan). All samples were prepared by placing a drop of primary sample on a holy carbon coated copper grid, which was then dried for 6 h at 80 °C in an oven. Prior to the sample preparation, an aqueous suspension of NPs was sonicated for several minutes. FT-IR spectra were recorded on a Perkin Elmer Spectrum 100 FT-IR spectrometer (FT-IR, Perkin Elmer, Waltham, MA, USA) in transmittance mode in the 400–4000 cm^−1^ range. The aqueous suspension of NPs was centrifuged for 15 min at 10,000 rpm. The NPs were washed three times with water to remove unbound PE. The washed sample was dried in an oven and mixed and pressed with KBr to obtain pellets for FT-IR measurements. The zeta potential of the as-prepared NPs was measured on a zeta potential analyzer (Malvern Zetasizer (7.11) nano instrument (USA), using Zeta Plus software) at room temperature by suspending the nanoparticle in aqueous solution.

## 3. Results and Discussions

**Figure 1:**
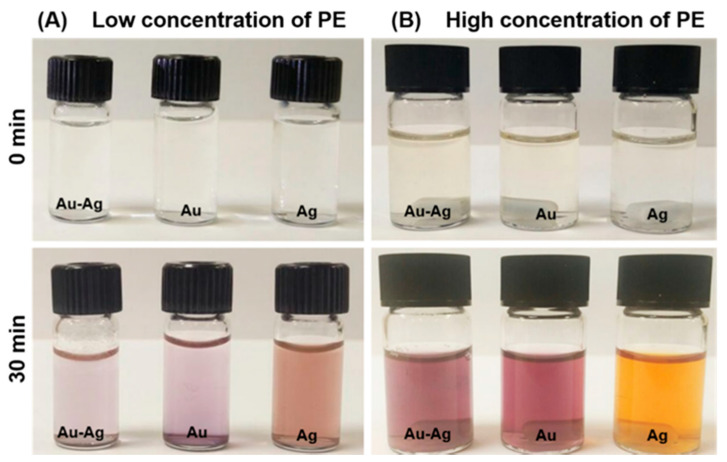
Ag, Au, and bimetallicalloy Au–Ag NPs with different concentrations of *P. undulata* PE; (**A**) lower concentration of PE and (**B**) higher concentration of PE.

Au, Ag, and Au–Ag bimetallic NPs with irregular morphologies (including triangular and hexagonal or quasi-elongated plates) were formed while using low concentrations of PE extracts. After adding the PE (low concentration), the color of the Au dispersion gradually changed to purple. The color of the Ag dispersion changed from colorless to brown. The color of a mixture of Au and Ag precursors changed to light purple (Figure 1A). For higher concentrations of PE, the Au, Ag, and Au–Ag NPs dispersions turned dark purple, dark yellow, and light purple, respectively (Figure 1B). The color change of the solutions indicated the reduction of Au and Ag ions to metallic counterparts.

**Figure 2:**
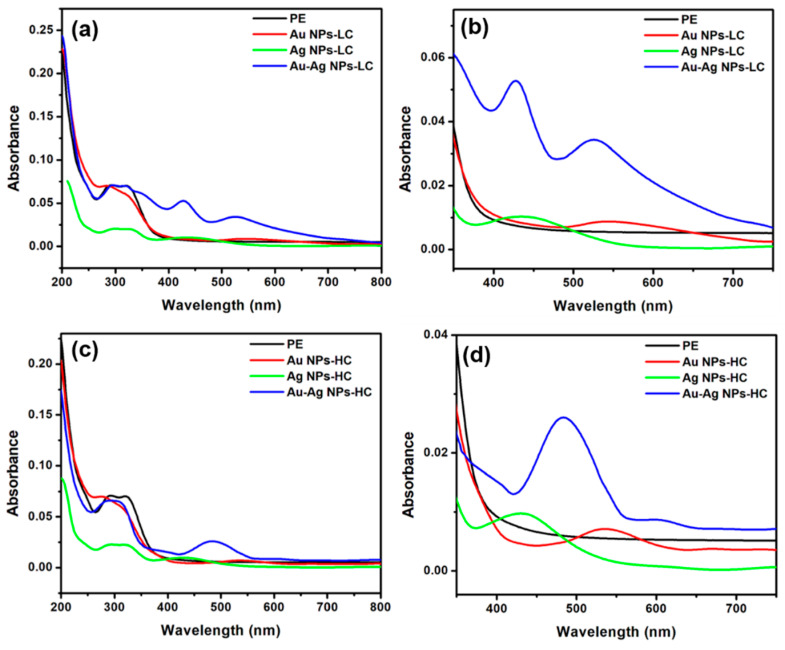
UV–vis spectra of Ag, Au, and Au–Ag bimetallic NPs with different concentrations of plant extract, i.e., (i) LC = low concentration of PE, (ii) HC = high concentration of PE. (**a**) UV–vis spectra of PE, Au, Ag, and Au–Ag phase segregated bimetallic NPs prepared with low concentration (LC) of plant extract (PE). (**b**) Zoom-in version of the surface plasmon bands related to the spectra shown in (**a**). (**c**) UV–vis spectra of PE, Au, Ag, and Au–Ag alloy bimetallic NPs prepared with high concentration (HC) of plant extract (PE). (**d**) Zoom-in version of the surface plasmon bands related to the spectra presented in (**c**).

NPs formation was confirmed by UV–vis spectroscopy. Figure 2 shows the UV–vis spectra of PE, Au, Ag, and Au–Ag NPs for different concentrations of PE. The UV–vis spectra of the Au and Ag NPs prepared with a low concentration of PE shows characteristic surface plasmon bands centered at ~548 nm and ~440 nm, respectively (Figure 2a red and green lines). The spectrum of the Au–Ag bimetallic NPs showed two distinct absorption bands at ~528 nm and ~430 nm corresponding to both Au and Ag NPs (Figure 2a, blue line). The presence of the two surface plasmon bands related to both Au and Ag NPs indicated the formation of phase segregated Au and Ag NPs. To better visualize these absorption bands, the zoom-in portion of the spectra are given in Figure 2b. For higher concentrations of PE, the spectral features and the positions of the absorption bands characteristic for Au, Ag, and Au–Ag NPs changed slightly compared to the samples prepared with low PE concentrations. The absorption band of the Au and Ag NPs were blue-shifted to ~430 nm and ~540 nm, respectively (Figure 2c, red and green lines). The blue shift in the surface plasmon band could be due to small size of the Au and Ag NPs, synthesized using higher concentrations of PE [35]. It is worth mentioning that the surface plasmon band of Au–Ag bimetallic NPs synthesized in the presence of a higher concentration of PE showed one broad absorption band centered at ~490 nm (Figure 2b, blue line). The absence of the separate surface plasmon bands of Au and Ag and the appearance of single broad band indicate the formation of Au–Ag alloy NPs [36]. The zoom-in version of the corresponding plasmon bands are shown in Figure 2d. In addition, a small absorption peak also appeared at ~600 nm in the UV spectrum of Au–Ag alloy NPs (cf. Figure 2d, blue line). Possibly, this peak can be attributed to the incomplete conversion of individual Ag and Au NPs to their respective alloy. The peak might belong to the remaining individual gold NP, which is visible due to the larger content of Au used during the preparation of alloy NPs.

**Figure 3:**
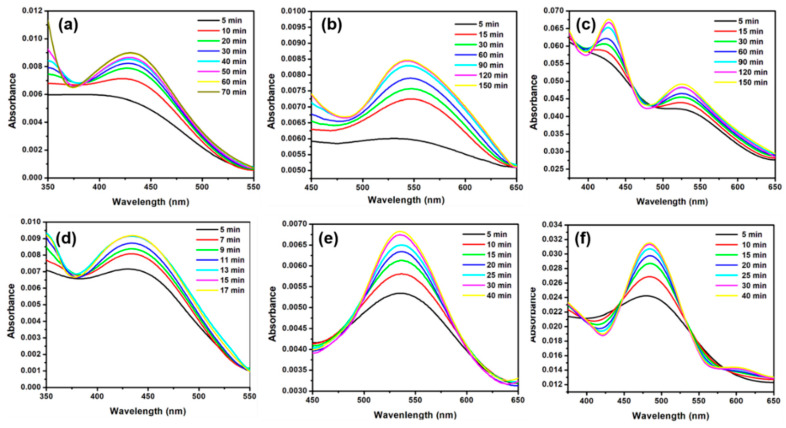
Kinetic analysis of the formation of Ag, Au, and Au–Ag bimetallic (phase separated/alloy) NPs prepared with different concentrations of PE. (**a**) Ag NPs, (**b**) Au NPs, and (**c**) Au–Ag NPs prepared using low concentrations of plant extract. (**d**) Ag, (**e**) Au, and (**f**) Au–Ag alloy NPs prepared using higher concentrations of plant extract.

To see the influence of concentration of PE on the rate of reaction, the kinetics for the formation of metal NPs was studied by monitoring the changes in the absorption band as a function of time (Figure 3). For low concentrations of PE, the absorption bands of Ag (Figure 3a), Au (Figure 3b), and Au–Ag NPs (Figure 3c) appeared after several minutes after adding the PE. The bands’ intensities gradually increased with time. After two hours, no further changes occurred for Au and Au–Ag NPs. The formation of the Ag NPs was complete after ~60 min. Notably, a slight shift towards lower wavelength is observed in the case of Ag NPs in Figure 3a, which may indicate the change in the size of NPs with increasing reaction time, since the UV peak is known to shift towards lower wavelength with decreasing size of NPs [37]. The longer reaction time in the case of Ag NPs compared to other samples may have affected the size of NPs leading to the shift in UV peak. When a high concentration of PE was used for the synthesis of metal NPs, the characteristic absorption bands for the metals appeared immediately after addition of PE. The intensities of these bands also increased rapidly with time. This shows a rapid reduction of Ag (Figure 3d) and Au (Figure 3e) ions for high PE concentrations and a fast formation of metal NPs. The intensity change of the absorption bands for Au and Au–Ag alloy NPs (Figure 3e,f) ceased after 30 min. In case of Ag, it stopped growing after 15 min (Figure 3d). This shows that when using a high concentration of PE, the formation of Au and Au–Ag alloy NPs was completed after 30 min, whereas the formation of Ag NPs was completed after 15 min (Figure 3d).

In general, the PE reductants often need elevated temperatures and long reaction times (several hours) for nanoparticle formation, which may hamper broad applications [38]. As an example, the formation of Ag NPs with *P. glutinosa* PE required 24 h at 90 °C [34]. In contrast, PE of *P. undulata* can form metal NPs at ambient temperature. The strong reducing properties of *P. undulata* can be attributed to the increased presence of phenolic phytomolecules such as kaempferol, quercetin, caffeic acid, dihydrokaempferol, and their glycosides [32]. Interestingly, these types of phytomolecules are known to possess efficient reducing abilities and have been detected in appreciable amounts in *P. undulata,* whereas *P. glutinosa* does not contain such phenolic phytomolecules [31,39,40]. This suggests that these phytomolecules (Figure S2) are involved in metal reduction. A confirmation of this hypothesis requires a comparative phytochemical investigation of both plants, which is currently under study.

*P. undulata* PE rendered very stable Au, Ag, and Au–Ag NPs in aqueous solution, because its phytochemical constituents acted not only as reductants but also as capping agents for surface functionalization of these NPs. This was confirmed by a comparison of the FT-IR spectra of as-prepared Au, Ag, and Au–Ag NPs and pure PE. The FT-IR spectra are provided in the Supporting Information (Figure S3). The IR spectra of purified metal NPs (Au, Ag, and Au–Ag NPs) and PE closely resembled each other, which confirmed the presence of phytomolecules as surface ligands on the NP surface. Analysis of these spectra showed the presence of phenolic and flavonoid groups, which are responsible for NP formation [41]. The IR spectrum of PE exhibited a broad absorption between 3791 and 3440 cm^−1^ and sharp bands at ~2929 and 2850 cm^−1^, which were attributed to the stretches of the O–H and C–H groups. Characteristic bands for C−H deformations and C−C and C−O stretches were present at 1753, 1620, and 1405 cm^−1^, respectively. The C−O stretches of alcohol and ether groups appeared at 1264 and 1059 cm^−1^, respectively. Most of these bands appeared as well in the IR spectra of as-prepared NPs with slight shifts and reduced intensities, which indeed confirmed their dual role for reduction and surface functionalization.

### 3.1. Effect of Concentration of PE on the Size and Morphology of NPs

**Figure 4:**
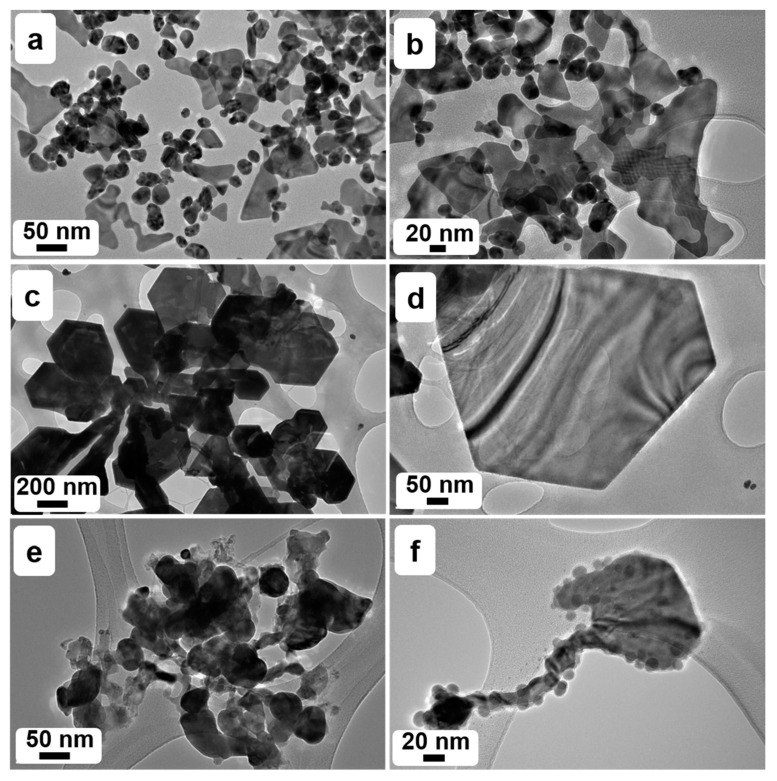
TEM images showing the morphologies of Au (**a**,**b**), Ag (**c**,**d**), and phase-separated Au–Ag (**e**,**f**) NPs using low concentrations of PE.

**Figure 5:**
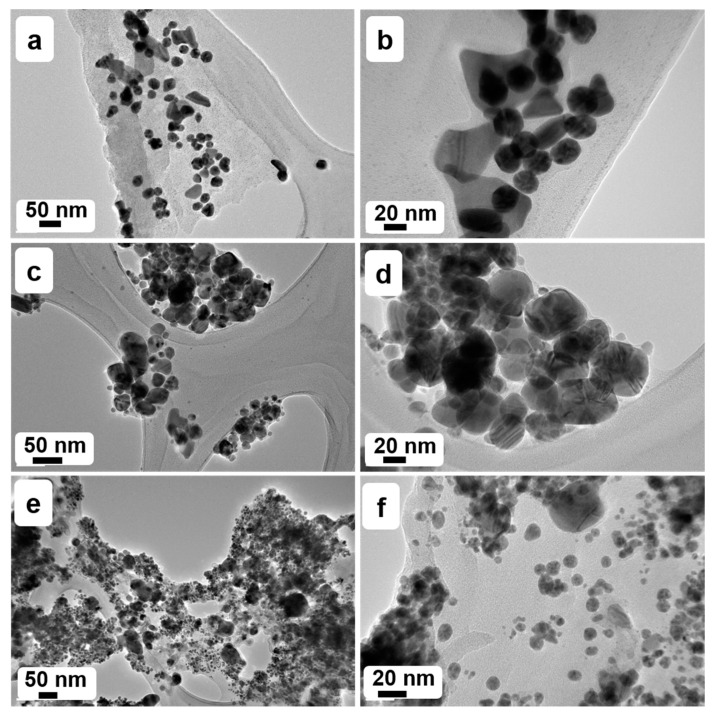
TEM images showing the morphologies of Au (**a**,**b**), Ag (**c**,**d**), and Au–Ag alloy (**e**,**f**) NPs using high concentrations of PE.

The PE concentration had a direct influence on the rate of reduction and particle morphology. For low concentrations of PE, the rate of reduction was low, resulting in slow nucleation and growth processes. In the absence of enough surface stabilizing ligand (low concentration of PE), the nuclei coalesced, resulting in the growth of anisotropic, irregular shaped NPs (including triangular and hexagonal or quasi-elongated plates (Figure 4a-e)). For Au–Ag mixtures, the reduction of the Au^3+^ component was faster than that of Ag^+^. This led to the growth of separate domains, where the domains were still attached to each other through the (multifunctional) surface ligands (Figure 4f). For high concentrations of PE (Figure 5), the reaction rate was faster, and at the same time, there was enough concentration of ligand available to stabilize the surface energy of the nuclei, resulting in quasi-isotropic NPs. This was also indicated by the UV–vis spectra presented in Figure 3f. The composition of all NPs was confirmed by EDX, and the respective EDX spectra are provided in the Supporting Information (Figures S4–S9).

Indeed, the dual role of PE was further confirmed by measuring the zeta potential of as-prepared NPs at pH = 7. The phytomolecules-based stabilization of metallic NPs typically renders a negative charge on the surface of NPs [42]. In this case, the zeta potential values of all the NPs were determined to be negative and were in the range of −9 to −19 mV at pH = 7. This gave additional evidence that the as-prepared metallic NPs were indeed capped by phytomolecules of PE. Furthermore, the zeta potential data also revealed that with increasing concentration of PE, the stability of the NP suspensions was also slightly increased. For instance, at lower concentrations of PE, smaller negative values of zeta potential of −9.93, −15.5 and −17.4 mV were obtained in the case of Au, Ag, and Au–Ag NPs, respectively, whereas the Au, Ag, and Au–Ag NPs obtained by using higher concentrations of PE demonstrated slightly larger negative values, such as −18.3, −16.2, and −18.4, respectively. The zeta potential figures are provided in the Supporting Information (Figures S10–S15). This indicated that with the increasing concentration of PE, the stability of the NP dispersion also increased due to increased adsorption of phytomolecules on the surface of the NPs. The Au and Ag monometallic NPs were irregular or anisotropic in shape; therefore, it was not possible to measure particle sizes. However, the Au–Ag alloy NPs using higher concentrations of PE were more regular in shape. The particles size histogram showed that the majority of the Au–Ag alloy NPs synthesized using higher concentrations of PE were in the range of 5 to 12 nm (with few exceptions of bigger NPs). The histograms are shown in Figure S16b. The Au–Ag bimetallic NPs synthesized using low concentrations of PE were anisotropic with attached spherical small particles. These small particle size histograms were constructed showing that these smaller domains were in the range of 10–20 nm (Figure S16a). 

### 3.2. Catalytic Application

The catalytic activities of the as-prepared Au, Ag, and Au–Ag alloy NPs were probed for the reduction of 4-nitrophenol (4-NPh). The reduction of 4-NPh to 4-aminophenol (4-APh) in the presence of NaBH_4_ is thermodynamically favorable. However, metal catalysts are needed to overcome the kinetic barrier [43,44] to transfer the electron from the BH_4_^−^ donor to the 4-NPh acceptor. The catalytic reactions were performed in a standard quartz cuvette (3 mL volume, 10 mm diameter), as described elsewhere [18]. A 2 mM solution of 4-NPh and 0.03 M solutions of NaBH_4_ were prepared separately, each in 10 mL of water. From these solutions, 0.3 mL of 4-NPh and 1 mL of NaBH_4_ were added into 1.4 mL of DI water. The solution was mixed thoroughly followed by the addition of 0.3 mL of a solution of the as-obtained NPs (Au/Ag/Au–Ag).

**Figure 6:**
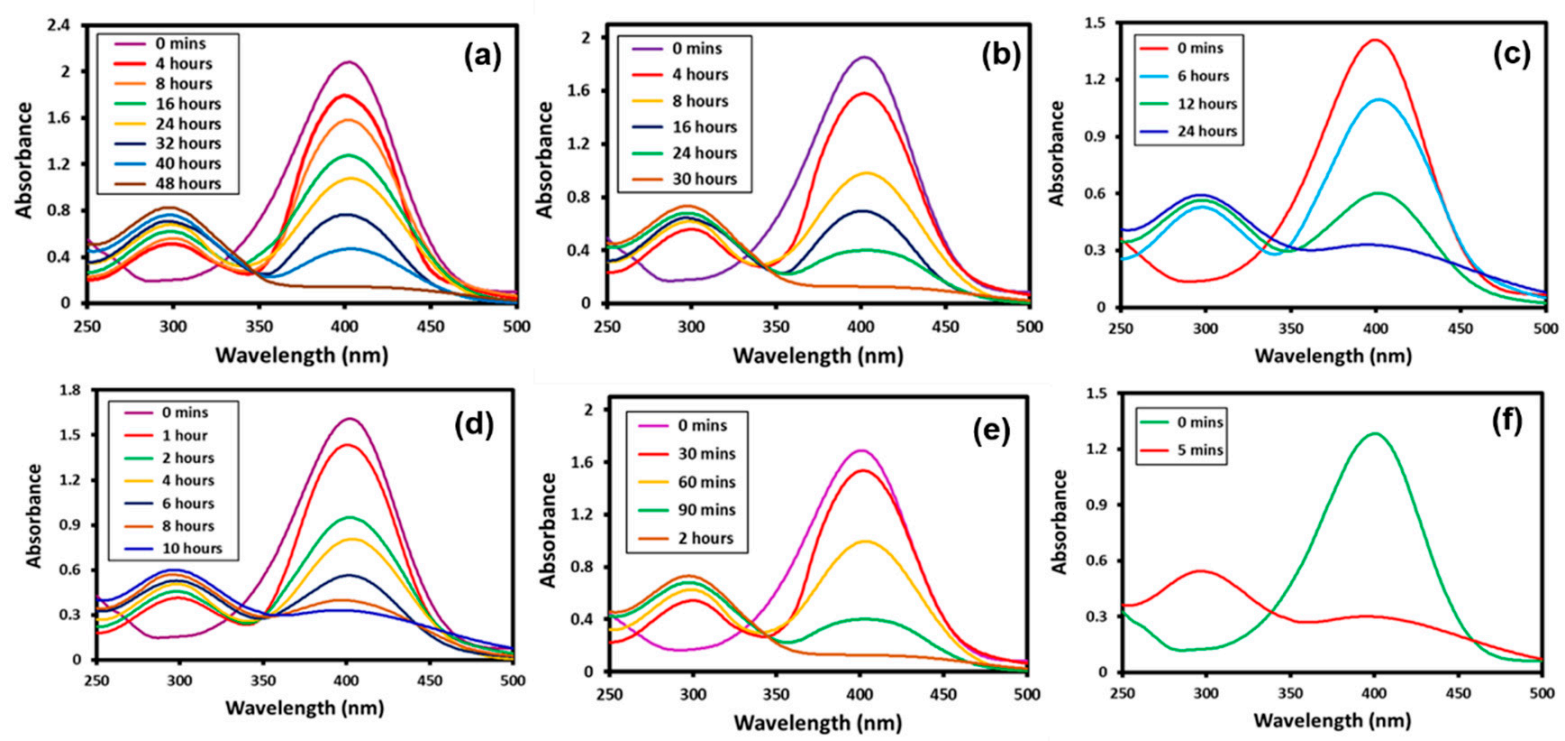
UV–Vis spectra showing the catalytic reduction of 4-nitrophenol using Au, Ag, and Au–Ag alloy NPs: (**a**–**c**) results of catalytic reduction of 4-NPh to 4-APh with Ag, Au, and Au–Ag NPs prepared using low concentrations of PE and (**d**–**f**) using Ag, Au, and Au–Ag NPs prepared using higher concentrations of PE.

The reduction of 4-NPh by NaBH_4_ in the presence of the NPs catalysts was monitored by UV–Vis spectroscopy from the fading of the yellow color of the 4-NPh substrate. The 4-NPh showed a characteristic absorption band at ~317 nm, which shifted to 400 nm due to the formation of 4-nitrophenolate ion in the presence of NaBH_4_ [45]. Therefore, the reduction of 4-NPh can be monitored by the intensity change of the band of 4-nitrophenolate at 400 nm. The reduction of 4-NPh was performed using as-prepared metal (Au, Ag, and Au–Ag) NPs with different morphologies obtained using both low as well as high concentrations of PE (Figure 6). The reduction of 4-NPh in the presence of the Au, Ag, and Au–Ag NPs is evident from the decrease in the absorbance at 400 nm (4-NPh) and the simultaneous increase of 4-aminophenolate band at 300 nm. In the absence of NPs, no change in the absorption band centered at 400 nm was observed. Representative absorption spectra of all catalytic reactions with Au, Ag, and Au–Ag NPs showed that the reaction rate decreased in the order Ag < Au < Au–Ag. The NPs obtained for high PE concentrations showed higher catalytic efficiency than NPs prepared at low concentrations of PE. The reduction of 4-NPh with NP catalysts prepared at low PE concentrations required several hours to days (Au–Ag: ~24 h, Au: ~30 h, Ag: >48 h: (Figure 6a,b,c)). With NP catalysts prepared using higher concentrations of PE, the reduction was completed significantly in less time, i.e., Au–Ag: ~5 min, Au: ~2 h, Ag: >10 h (Figure 6d,e,f). In order to test the importance of the catalyst, a blank reaction was performed using 0.3 mL of 4-NPh, 1 mL of NaBH_4_, and 1.4 mL of DI water without adding any catalyst. No change in the characteristic absorption peak of 4-NPh was observed, as seen in Figure S17a. In addition, to investigate the effect of phytoconstituents attached on the surface of catalysts on the reduction process, another blank reaction was performed using a minute amount of PE. The blank experiment using PE was performed by using 0.3 mL of 4-NPh, 1 mL of NaBH_4_, and 1.4 mL of DI water. The solution was mixed thoroughly followed by the addition of 0.3 μL of PE from a previously prepared stock solution (10 mg of PE in 1 mL of water). Notably, PE (without catalysts) also did not induce the reduction as the absorption peak of 4-NPh did not show any change (Figure S17b). Although, polyphenols of PE are capable of inducing the reduction process, we used, however, a very minute amount of PE in this reaction, which was not sufficient to induce the reduction process. Therefore, from this experiment it is clear that the phytomolecules attached on the surface of catalysts as ligand do not play a significant role in the reduction of 4-NPh due to their minute quantity 

For Au–Ag alloy NPs, the reduction of 4-NPh started instantly, and the reaction was completed after only 5 min (Figure 6f). This confirms previous results that the activity of Au–Ag alloy NPs is higher than that of elemental Au or Ag NPs [46]. The enhanced catalytic activity of the alloy NPs is attributed to structural effects on the electronic states [47]. Au–Ag NPs contain cavities that confine reactants by acting as “nano-reactor cages”. The active surface sites in the voids of the Au–Ag alloys with edges or kinks are considered as key for the enhanced catalytic activity and selectivity [48]. In addition, bimetallic NPs provide Au–Ag interfaces with special electronic effects that facilitate a strong adsorption of the reactants to the catalyst surface, which eventually enhances the catalytic efficiency. Earlier studies showed that higher catalytic activity occurs near Au sites where the NPs surface changes from a segregated Au surface to a homogeneous alloy composition [49]. The surface of the Au–Ag samples contains mostly gold, whereas Ag appears only in minor amounts. Still, the presence of Ag is essential and enhances the charge/electron transfer to the 4-NPh substrate during the reduction because of the lower electronegativity of Ag compared to Au.

Notably, Au–Ag alloy NPs prepared by using different concentrations of PE exhibited varied catalytic activities. For instance, alloy NPs obtained at higher concentrations of PE facilitated the reduction of 4-NPh within a few minutes (cf. Figure 6f), whereas a similar reaction required several hours (cf. Figure 6c) when Au–Ag alloy NPs prepared at low concentrations of PE were used. Typically, one of the most important factor which governs the efficiency of nanocatalysts during the reduction of 4-NPh is the morphology of the nanocatalyst, since a well-tuned morphology with defined facets tends to increase the surface area of a nanocatalyst, which in turn enhances the active site of the catalyst in the reaction medium, and thus has a direct effect on its catalytic activity [50]. Therefore, in this case, Au–Ag alloy NPs obtained at higher concentrations of PE demonstrated higher catalytic activity towards the reduction of 4-NPh due to the well-defined spherical shaped morphology and smaller size (Figure 5) of the nanocatalyst as compared to Au–Ag bimetallic NPs synthesized using low concentrations of PE (Figure 4). On the other hand, the inefficient catalytic activity of Au–Ag alloy NPs was attributed to the ill-defined irregular shapes of NPs that were obtained by using low concentrations of PE. 

## 4. Conclusions

The fast synthesis of Au, Ag, and Au–Ag segregated or alloy NPs at room temperature was achieved with PE of *P. undulata* as reducing and stabilizing agent. It is significantly faster than using extracts of some other plants as reducing agent. The concentration of PE had a significant effect on the morphology and composition of the NPs. Isotropic Au–Ag alloy NPs and well-defined elemental Au and Ag NPs were obtained at high PE concentrations. Nanoparticles with irregular morphology, domain segregated bimetallic NPs, and elemental Au and Ag NPs were formed at low PE concentrations. Phytochemical and IR spectroscopy analysis of the particles showed that the PE contains large shares of flavonoids and polyphenols, which are responsible for the rapid reduction of metal precursors. Moreover, these molecules serve as surface ligands that stabilize the metal and alloy particles. Catalytic reduction of 4-NPh to 4-APh in the presence of as-prepared metal and alloy NPs showed very high activity for Au–Ag alloy NPs prepared at high PE concentrations. Elemental Ag NPs displayed the lowest catalytic activity, while elemental Au particles have shown intermediate performance.

## Supplementary Materials

The following are available online at https://www.mdpi.com/2079-4991/10/9/1885/s1, Figure S1: (a) UV spectra of Au, Ag and Au-Ag alloy nanoparticles, (b,c,d) high resolution TEM images of Au, Ag, and Au-Ag alloy nanoparticles, Figure S2: Chemical structures of some of the phytomolecules present in the *P. undulata* plant extract, Figure S3: FT-IR spectra of pure PE and Ag, Au and Au-Ag alloy NPs prepared with a high (1:2) concentration of PE, Figure S4: Energy dispersive X-ray spectrum of Au-Ag-alloy NPs using higher concentration (AuAg-HC) of PE, Figure S5: Energy dispersive X-ray spectrum of Au-Ag-bimetallic NPs using low concentration (AuAg-LC) of PE, Figure S6: Energy dispersive X-ray spectrum of Ag NPs using higher concentration (Ag-HC) of PE, Figure S7: Energy dispersive X-ray spectrum of Ag NPs using low concentration (Ag-LC) of PE, Figure S8: Energy dispersive X-ray spectrum of Au-NPs using higher concentration (Au-HC) of PE, Figure S9: Energy dispersive X-ray spectrum of Au-NPs using low concentration (Au-LC) of PE, Figure S10: Zeta potential analysis of Au NPs using low concentration of plant extract, Figure S11: Zeta potential analysis of Ag NPs using low concentration of plant extract, Figure S12: Zeta potential analysis of Au-Ag NPs using low concentration of plant extract, Figure S13: Zeta potential analysis of Au NPs using high concentration of plant extract, Figure S14: Zeta potential analysis of Ag NPs using high concentration of plant extract, Figure S15: Zeta potential analysis of Au-Ag NPs using high concentration of plant extract, Figure S16: Particle size distribution graph of Au-Ag NPs using low concentration of plant extract and Au-Ag NPs using high concentration of plant extract, Figure S17: UV spectra of 4-NP of blank reaction performed (a) in the absence of catalyst, (b) using a very minute amount of plant extract.
