# Supplementary material for: Synthesis of Au, Ag, and Au–Ag Bimetallic Nanoparticles Using Pulicaria undulata Extract and Their Catalytic Activity for the Reduction of 4-Nitrophenol
Source: Nanomaterials (Basel). 2020 Sep 20;10(9):1885. doi: 10.3390/nano10091885 (PMC7559643; doi:10.3390/nano10091885)
Supplement: Supplementary file 1 [file nanomaterials-10-01885-s001.pdf]

nanomaterials

MDPI

Supplementary File

Synthesis of Au, Ag, and Au–Ag Bimetallic Nanoparticles Using *Pulicaria undulata* Extract and Their Catalytic Activity for the Reduction of 4-Nitrophenol

Merajuddin Khan [1], Khaleel Al-hamoud [1], Zainab Liaqat [2], Mohammed Rafi Shaik [1], Syed Farooq Adil [1], Mufsir Kuniyil [1], Hamad Z. Alkhathlan [1], Abdulrahman Al-Warthan [1], Mohammed Rafiq H. Siddiqui [1], Mihail Mondeshki [2], Wolfgang Tremel [2,*], Mujeeb Khan [1,*] and Muhammad Nawaz Tahir [3,*]

[1] Department of Chemistry, College of Science, King Saud University, P.O. Box 2455, Riyadh 11451, Saudi Arabia; mkhan3@ksu.edu.sa (M.K.); khaleel727244@gmail.com (K.A.-h.); mrshaik@ksu.edu.sa (M.R.S.); sfadil@ksu.edu.sa (S.F.A.); mkuniyil@ksu.edu.sa (M.K.); khathlan@ksu.edu.sa (H.Z.A.); awarthan@ksu.edu.sa (A.A.-W.); rafiqs@ksu.edu.sa (M.R.H.S.)

[2] Department Chemie, Johannes Gutenberg Universität of Mainz, Duesbergweg 10-14, D-55128 Mainz, Germany; lizainab@uni-mainz.de (Z.L.); mondeshk@uni-mainz.de (M.M.)

[3] Department of Chemistry, King Fahd University of Petroleum & Minerals, P.O. Box 5048, Dhahran 31261, Saudi Arabia

* Correspondence: tremel@uni-mainz.de (W.T.); kmujeeb@ksu.edu.sa (M.K.); muhammad.tahir@kfupm.edu.sa (M.N.T.); Tel.: +966-114-670-439 (M.K.)

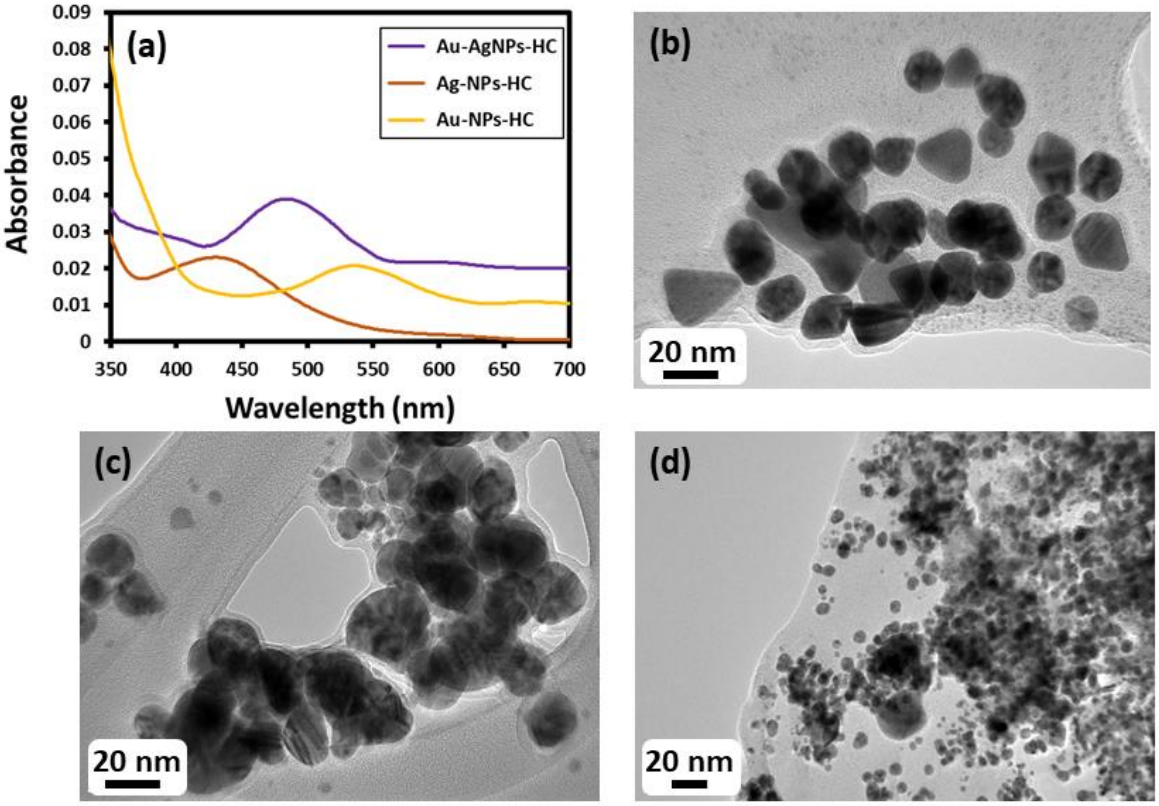

Figure S1: To confirm the reproducibility of experiments, Ag, Au and Au-Ag alloy nanoparticles has been reproduced using high concentration of plant extract from same batch. All the experiments were performed by the same procedure given in the experimental section 2.3 of the main-text. (a) UV spectra of Au, Ag and Au-Ag alloy nanoparticles, (b, c, d) high resolution TEM images of Au, Ag, and Au-Ag alloy nanoparticles.

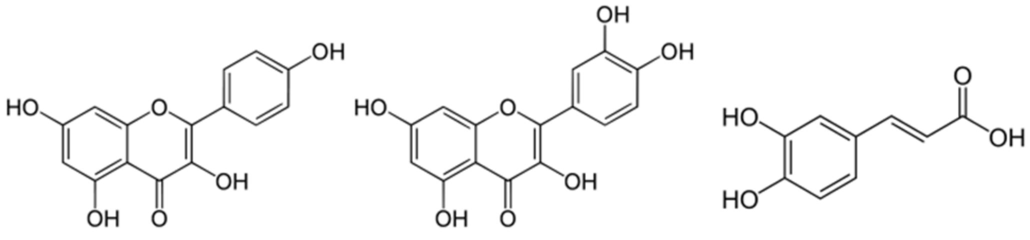

Kaempferol **Quercetin** **Caffeic acid**

Figure S2: Chemical structures of some of the phytomolecules present in the *P. undulata* plant extract.

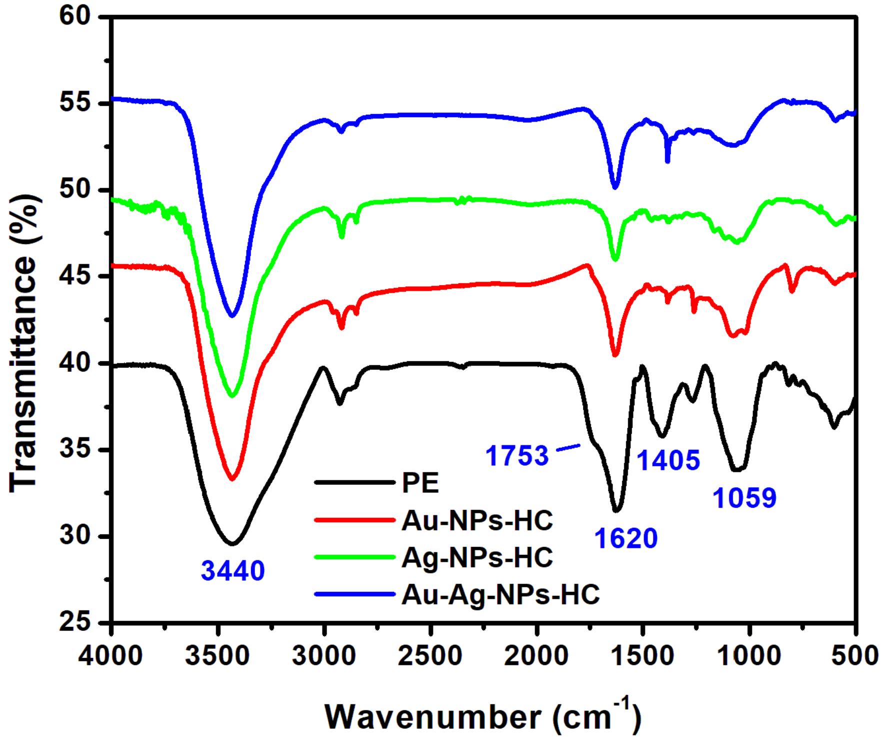

Figure S3: FT-IR spectra of pure PE and Ag, Au and Au-Ag alloy NPs prepared with a high (1:2) concentration of PE.

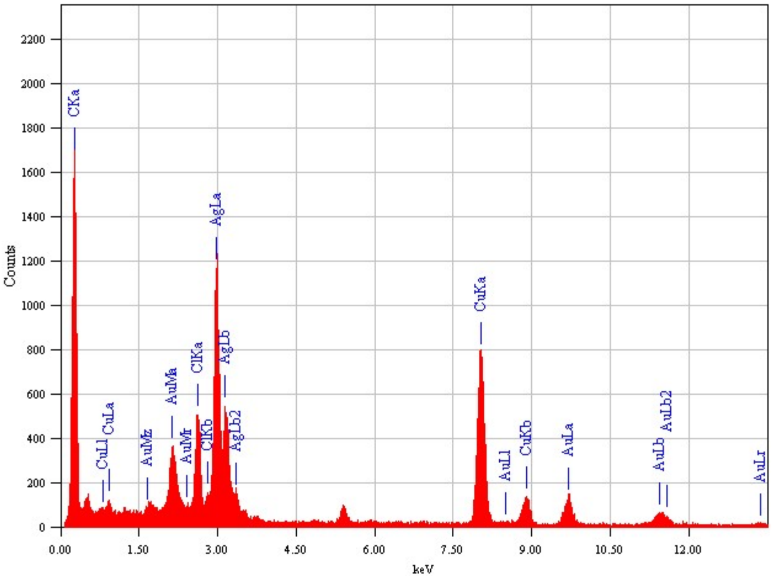

Figure S4: Energy dispersive X-ray spectrum of Au-Ag-alloy NPs using higher concentration (AuAg-HC) of PE.

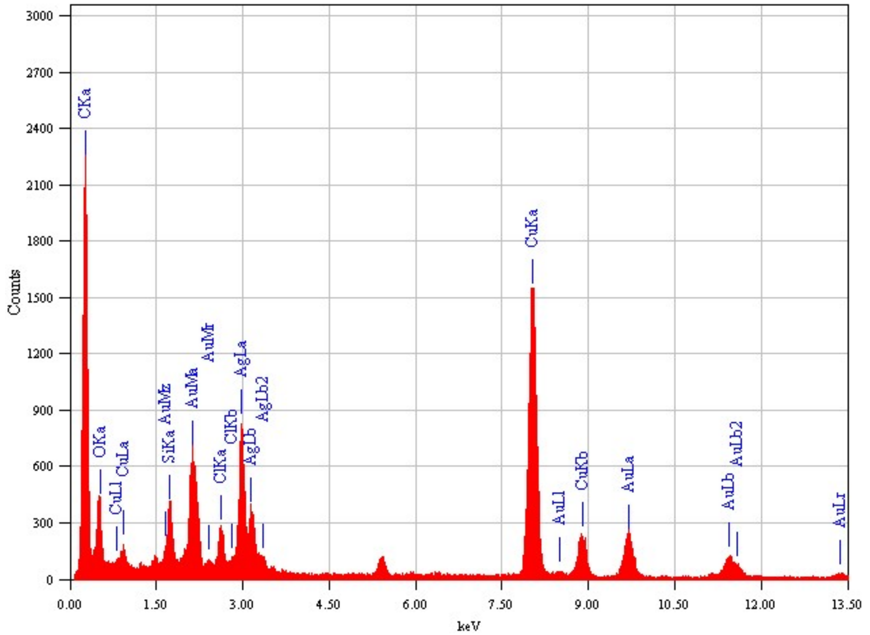

Figure S5: Energy dispersive X-ray spectrum of Au-Ag-bimetallic NPs using low concentration (AuAg-LC) of PE.

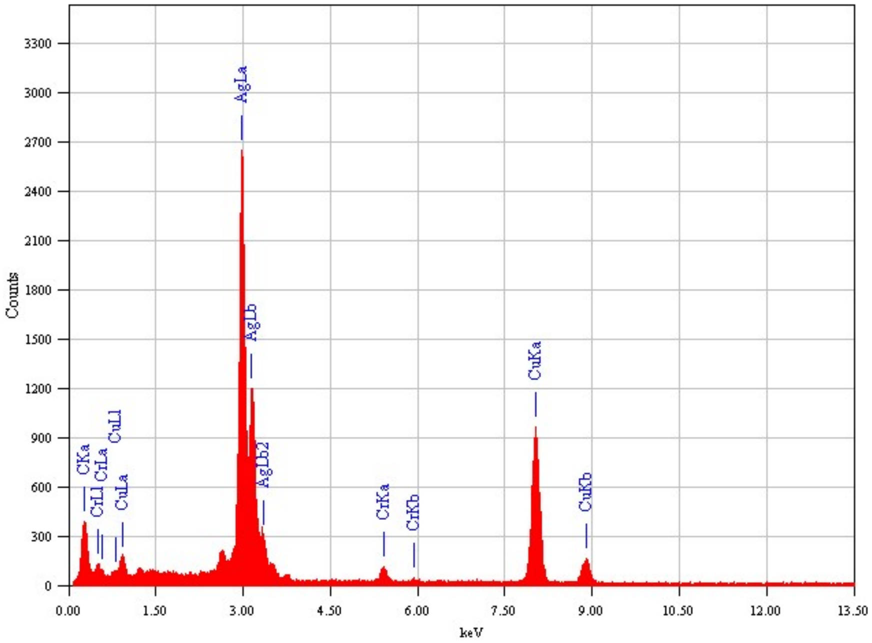

Figure S6: Energy dispersive X-ray spectrum of Ag NPs using higher concentration (Ag-HC) of PE.

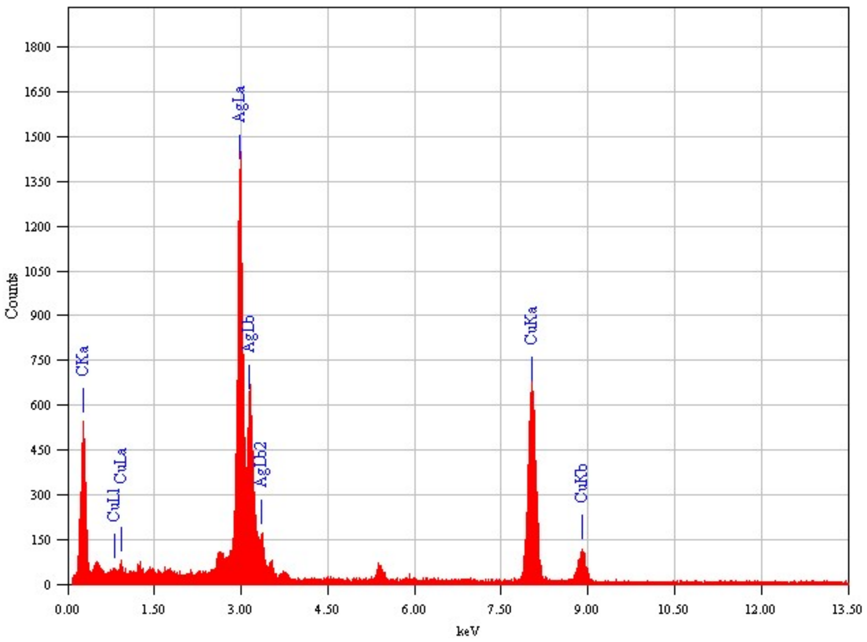

Figure S7: Energy dispersive X-ray spectrum of Ag NPs using low concentration (Ag-LC) of PE.

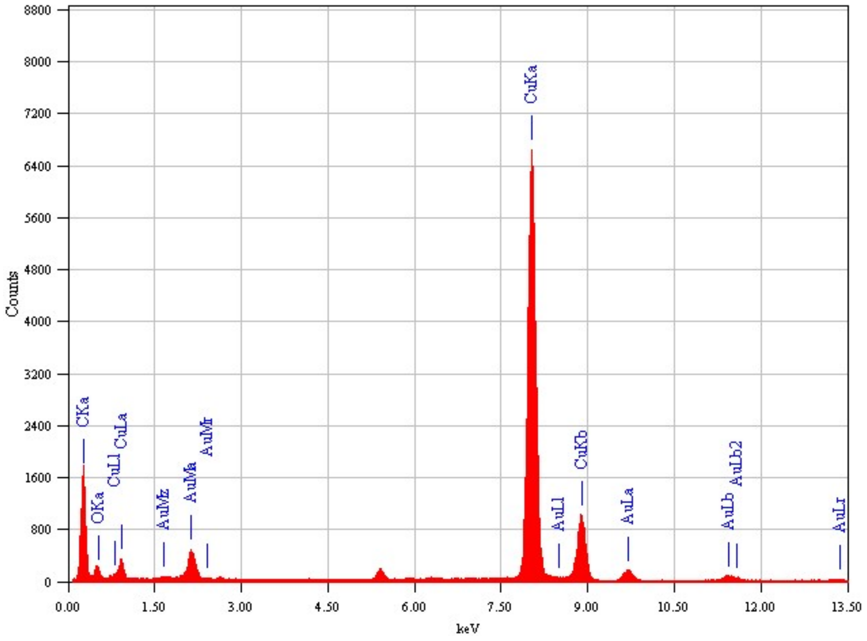

Figure S8: Energy dispersive X-ray spectrum of Au-NPs using higher concentration (Au-HC) of PE.

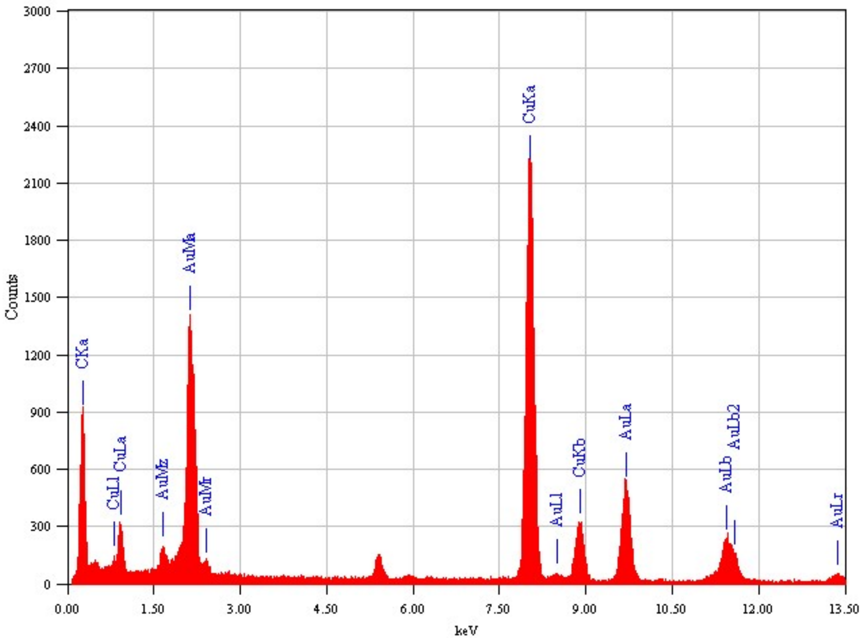

Figure S9: Energy dispersive X-ray spectrum of Au- NPs using low concentration (Au-LC) of PE.

Results

			Mean (mV)	Area (%)	St Dev (mV)
Zeta Potential (mV):	-9.93	Peak 1:	-9.93	100.0	6.64
Zeta Deviation (mV):	6.64	Peak 2:	-40.1	0.0	0.581
Conductivity (mS/cm):	0.491	Peak 3:	0.00	0.0	0.00
Result quality :	Good				

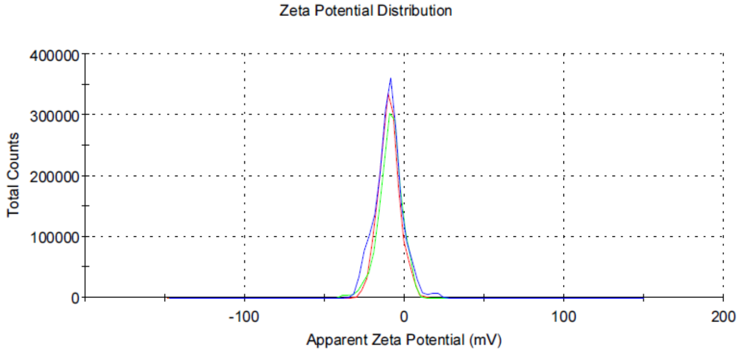

Figure S10: Zeta potential analysis of Au NPs using low concentration of plant extract.

Results

			Mean (mV)	Area (%)	St Dev (mV)
Zeta Potential (mV):	-15.5	Peak 1:	-16.9	87.0	6.55
Zeta Deviation (mV):	8.32	Peak 2:	-0.158	12.6	2.89
Conductivity (mS/cm):	0.172	Peak 3:	-43.3	0.4	2.03
Result quality :	Good				

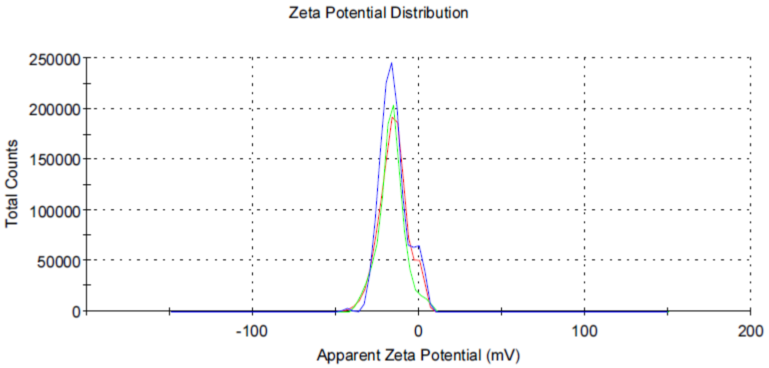

Figure S11: Zeta potential analysis of Ag NPs using low concentration of plant extract.

Results

			Mean (mV)	Area (%)	St Dev (mV)
Zeta Potential (mV):	-17.4	Peak 1:	-17.4	100.0	7.75
Zeta Deviation (mV):	7.75	Peak 2:	0.00	0.0	0.00
Conductivity (mS/cm):	0.239	Peak 3:	0.00	0.0	0.00
Result quality :	See result quality report				

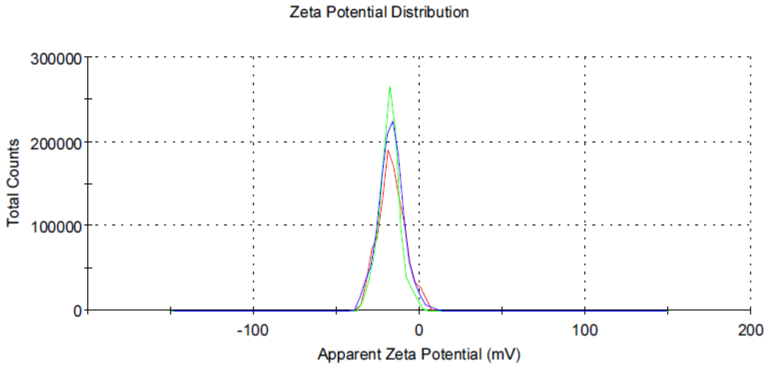

Figure S12: Zeta potential analysis of Au-Ag NPs using low concentration of plant extract.

Results

			Mean (mV)	Area (%)	St Dev (mV)
Zeta Potential (mV):	-18.3	Peak 1:	-17.1	93.3	9.31
Zeta Deviation (mV):	11.3	Peak 2:	-42.4	4.7	3.96
Conductivity (mS/cm):	0.312	Peak 3:	-55.7	1.3	3.65
Result quality :	See result quality report				

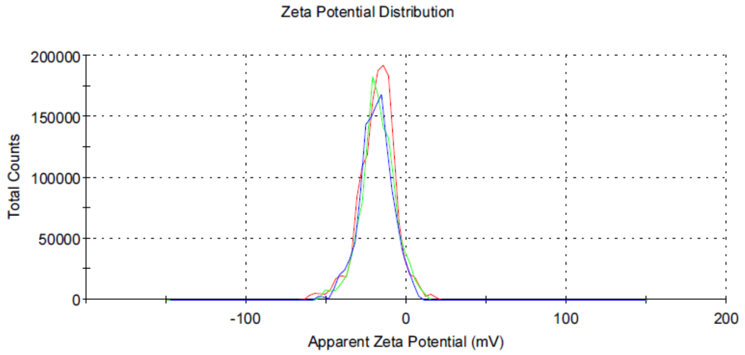

Figure S13: Zeta potential analysis of Au NPs using high concentration of plant extract.

Results

			Mean (mV)	Area (%)	St Dev (mV)
Zeta Potential (mV):	-16.2	Peak 1:	-16.2	100.0	6.70
Zeta Deviation (mV):	6.70	Peak 2:	0.00	0.0	0.00
Conductivity (mS/cm):	0.129	Peak 3:	0.00	0.0	0.00
Result quality :	Good				

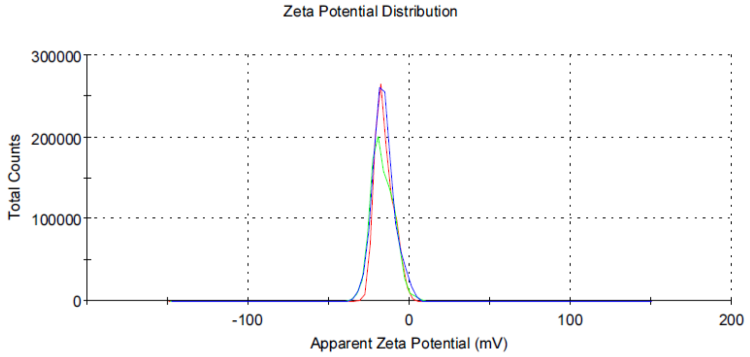

Figure S14: Zeta potential analysis of Ag NPs using high concentration of plant extract.

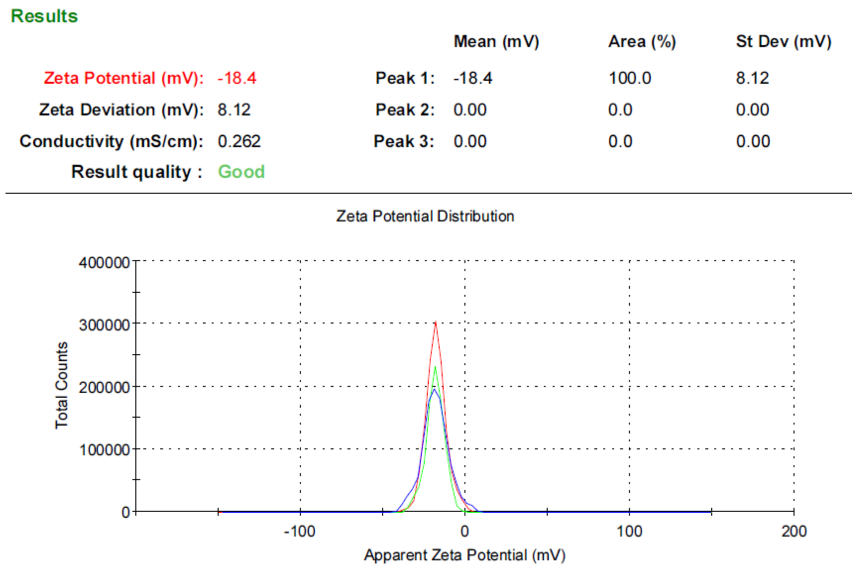

Figure S15: Zeta potential analysis of Au-Ag NPs using high concentration of plant extract.

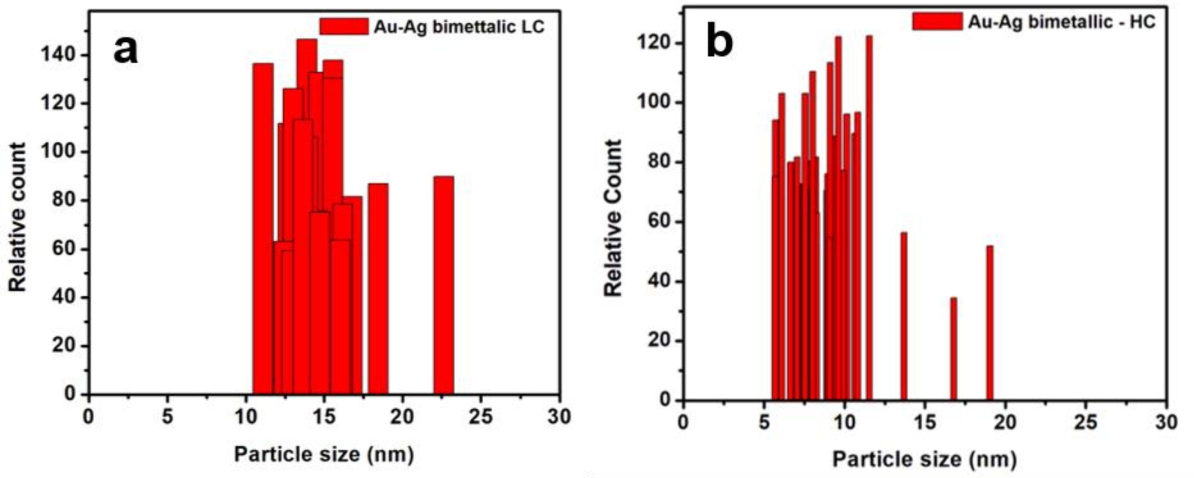

Figure S16: Particle size distribution graph of Au-Ag NPs using low concentration of plant extract and Au-Ag NPs using high concentration of plant extract.

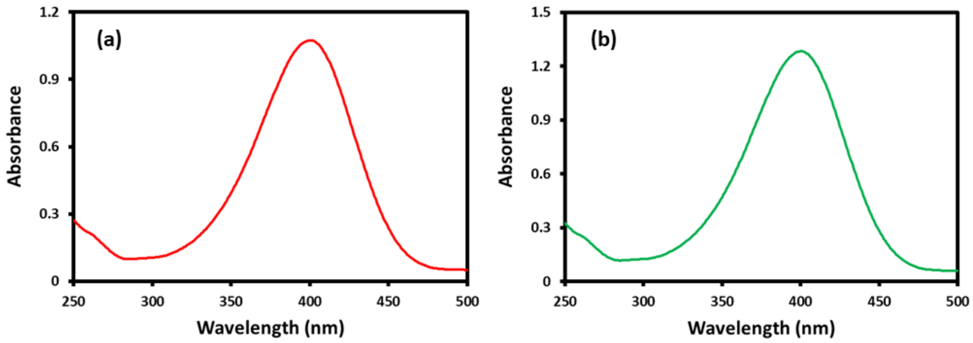

Figure S17: UV spectra of 4-NP of blank reaction performed (a) in the absence of catalyst, (b) using a very minute amount of plant extract.

.
